# Finite element modelling of sound transmission in the Weberian apparatus of zebrafish (*Danio rerio*)

**DOI:** 10.1098/rsif.2023.0553

**Published:** 2024-01-10

**Authors:** Jordi Marcé-Nogué, Juan Liu

**Affiliations:** ^1^ Department of Mechanical Engineering, Universitat Rovira i Virgili Tarragona, 43007 Tarragona, Catalonia, Spain; ^2^ Institut Català de Paleontologia, Universitat Autònoma de Barcelona, 08193 Cerdanyola del Vallès, Catalonia, Spain; ^3^ Department of Integrative Biology, University of California, Berkeley, Berkeley, CA 94720, USA; ^4^ University of California Museum of Paleontology, University of California, Berkeley, Berkeley, CA 94720, USA

**Keywords:** Weberian apparatus, sound conduction, finite element method, zebrafish, hearing

## Abstract

Zebrafish, an essential vertebrate model, has greatly expanded our understanding of hearing. However, one area that remains unexplored is the biomechanics of the Weberian apparatus, crucial for sound conduction and perception. Using micro-computed tomography (μCT) bioimaging, we created three-dimensional finite element models of the zebrafish Weberian ossicles. These models ranged from the exact size to scaled isometric versions with constrained geometry (1 to 10 mm in ossicular chain length). Harmonic finite element analysis of all 11 models revealed that the resonance frequency of the zebrafish's Weberian ossicular chain is approximately 900 Hz, matching their optimal hearing range. Interestingly, resonance frequency negatively correlated with size, while the ratio of peak displacement and difference of resonance frequency between tripus and scaphium remained constant. This suggests the transmission efficiency of the ossicular chain and the homogeneity of resonance frequency at both ends of the chain are not size-dependent. We conclude that the Weberian apparatus's resonance frequency can explain zebrafish's best hearing frequency, and their biomechanical characteristics are not influenced by isometric ontogeny. As the first biomechanical modelling of atympanic ear and among the few non-human ear modelling, this study provides a methodological framework for further investigations into hearing mechanisms and the hearing evolution of vertebrates.

## 1. Introduction

Zebrafish, a remarkable vertebrate model organism, has played a pivotal role in advancing our understanding of various aspects of hearing. Extensive research on the hearing system of zebrafish has contributed significantly to the fields of auditory genetics [1], epigenetics [2], synaptopathy [3], hair cell regeneration [4], ototoxicity [5] and beyond. The hearing capability of zebrafish has been evaluated at early developmental stages [6,7], entire life span [8–11], ageing [12] and comparisons of laboratory fish lines [13] using behavioural and electrophysiological auditory methods, which serve as fundamental baselines for further exploring of hearing mechanisms. It is widely accepted and demonstrated that Weberian apparatus of zebrafish, like any other otophysan fishes, contribute to their high hearing sensitivities and large frequency range compared to non-otophysan fishes [9,14,15]. However, the Weberian apparatus, the functional analogue of middle ear in human, has not been part of the equation in hearing loss, form-function correlation, nor biomechanical studies.

The Weberian apparatus consists of a suite of ossicles and associated ligaments connecting the gas bladder (swim bladder) to the inner ear [16] in otophysan fishes including cypriniforms (carps, zebrafish, etc), siluriforms (catfishes), characiforms (characins) and gymnotiforms (South American knifefish). The ossicles develop from elements of the first three vertebrae [17–19]. The Weberian ossicles mechanically couple vibrations of the gas bladder induced by sound pressure to the fluid filled sac of the inner ear, allowing sound to be transmitted into inner ear. The Weberian ossicles of otophysan fishes are therefore functional analogues of the middle ear ossicles in terrestrial vertebrates.

In most otophysan fishes, there are four pairs of Weberian ossicles located bilaterally along the vertebrae column. From anterior to posterior, they are called claustrum, scaphium, intercalarium and tripus. Seminal works on morphology of Weberian ossicles have been performed by dissections and using light microscopes [16,20–23], and additional visualization using X-rays and measurements using callipers [24], as well as illustration using camera lucida and thin sections [25,26]. These works found that the tripus contacts the tunica externa of the anterior chamber of the gas bladder, while the claustrum and scaphium are coupled to the inner ear fluid space (the atrium of the sinus impar). Recent works on morphology, ontogeny and development of Weberian apparatus have extensively used clearing and staining methods [27–29], as well as histological thin sections to investigate and capture the morphology of Weberian ossicles [30,31]. These classical yet prevalent methods are essential and accurate to observe and image morphology of Weberian ossicles. Digital imaging techniques like X-ray-based computed tomography (CT) have since revolutionized the study of the Weberian apparatus, enabling the creation of precise three-dimensional visualizations with calibration, orientation and spatial relationship [32].

Sound transmission of Weberian ossicles occurs through their biomechanical response to sound pressure oscillations or fluctuations in a range of frequencies, comparable to the biomechanical characteristics of human middle ear ossicles (see a review for biomechanical modelling of human hearing by De Paolis *et al.* [33]. Despite having relatively well-understood anatomy and general function [20], the hearing mechanism of Weberian apparatus has only been modelled mathematically [34]. The biomechanical behaviour of Weberian ossicles during performance of sound conduction is yet to be explored and validated. To understand the mechanism of sound transmission through Weberian apparatus, we investigated the biomechanical characteristics by modelling the vibration transmission through the ossicles using finite element (FE) methods.

Recent research using behavioural and electrophysiological hearing test have shown that fishes with Weberian apparatus (otophysans) have increased frequency range and sensitivity compared with those without [35–37]. Hearing ability of zebrafish during early development to maturity has been tested by multiple research groups using behaviour and electrophysiology methods. It has been shown that the formation of Weberian apparatus significantly increases hearing sensitivity [9,38]. However, after Weberian apparatus formed and functioned, there is disagreement in terms of whether the hearing threshold changes along size/age [8,10,12,38]. On the other hand, there is no doubt that the Weberian apparatus undergoes growth in tandem with the overall body growth, displaying either isometric or allometric patterns. The size of the Weberian apparatus also varies among different otophysan species. To simulate the function of Weberian apparatus through varied sizes, we created a theoretical ontogenetical series of Weberian ossicles with the size ranged from 1 to 10 mm, with the morphology constrained to be the same as our original finite element model. The biomechanical responses to sound pressure of Weberian ossicles were then compared after finite element analysis. The aims of this study are twofolds: (i) to explore the biomechanical behaviours of Weberian ossicles when transmitting sound pressure in the form of vibrations from the gas bladder to the inner ear and (ii) to investigate whether the sizes of the Weberian apparatus influence biomechanical behaviours and the accompanying hearing characteristics.

## 2. Material and methods

**Figure 1.**
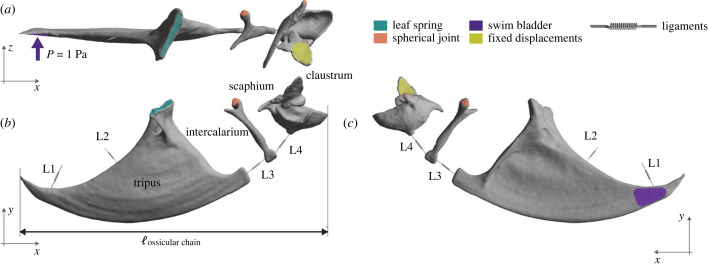
Finite element model of the Weberian apparatus in zebrafish. (*a*) Dorsal view, (*b*) lateral view and (*c*) medial view of Weberian ossicular chain with boundary conditions. Anterior of (*a*) and (*b*) to the right, and anterior of (*c*) to the left. Ligaments L1, L2, L3 and L4 are included as spring elements. Locations of the boundary conditions (fixed displacements) and the joints between ossicles and spines (leaf spring and spherical joints) are displayed. The ossicular chain length (ℓ_ossicular chain_) used to differentiate and scale the ontogenetic series is also drawn. *P* stands for simulated sound pressure.

In this study, we developed 11 finite element models of the zebrafish Weberian ossicle chain. The initial model is an accurate reconstruction derived from scaled image acquisition using micro-computed tomography (μCT) scans of an adult zebrafish. The original-size Weberian ossicle model has an ossicular chain length at 2.6 mm (figure 1*a,b*). Then, to test whether size affects the sound transmission function of Weberian ossicles, we built an isometric series which had the same morphology, but varied sizes. There were 10 theoretical models created with intervals at 1 mm and overall length ranging from 1 mm to 10 mm using the geometry of the original model. Ossicular length from 1 to 3 mm covered the possible sizes of the Weberian ossicle in zebrafish.

### 2.1. Materials

A specimen of an adult zebrafish (*Danio rerio*, KU22656 from Division of Ichthyology, Biodiversity Institute, University of Kansas (KU)) was scanned using Xradia Zeiss VersaXRM-520 at the Institute of Biotechnology, Cornell University (Ithaca, NY, USA). Specimen's standard length 25.9 mm, body depth 9.1 mm. The fish was stained in 0.3% phosphotungstic acid in 70% ethanol prior to CT scan following the procedure described by Metscher [39]. The scan speed was 1601 images/4S/exposure with radiation energy at 100 kV, 9 W, voxel size 4.64 μm. CT images are deposited at MorphoSource (morphosource.org, Media ID 000562737).

The image stack generated from CT scan was imported to the software Amira 6.0 (Thermo Scientific, Houston, TX, USA). We segmented the Weberian ossicles out from CT dataset using threshold selection and manual adjustment, and then created surface geometry models (.STL file) of the four Weberian ossicles: scaphium, claustrum, intercalarium and tripus. We then repaired, refined, and slightly smoothed the surface model using GeomagicWrap (3D Systems, v. 12, Rock Hill, SC, USA). Using the same software, the surface models were converted to CAD files following Marcé-Nogué *et al*. [40] for preparation of analysis. The geometry mesh file in STL format is deposited at MorphoSource (Media ID 000563167).

### 2.2. Model properties of the original model

A series of modal and harmonic response analyses were performed to evaluate the biomechanical performance of the bones of the Weberian apparatus using the finite element package ANSYS 17.1 (Ansys, Canonsburg, USA) and on a Dell Precision Workstation 7820 with 128 GB RAM, and 16 cores Intel(R) Xeon(R) Silver 4110 processor.

The material properties defined in these analyses were adopted from previous finite element analysis publications. Young's modulus was assumed as 16 GPa based on a two-needle indentation of rabbit ossicles [41]. The Poisson's coefficient is 0.3 [42–44]. Density of the bone is assigned with value 2.1 g cm^−^^3^, which is the density of human ear ossicles [45] and within the range of bone from closely related species (roach, carp and dace) of zebrafish [46].

The model was meshed using the ANSYS mesh module with an adaptive mesh of hexahedral elements [47]. The mesh of the model was approximately 230 000 elements, which is considered accurate for computing displacements. In a sensitivity test of increasing and decreasing the mesh number by 50%, the results of the following analyses were consistent.

### 2.3. Ligaments and connections between bones in the original model

Four ligaments (figure 1*b*) that play functional role in the Weberian apparatus have been described and illustrated by Alexander [20] and Finneran & Hastings [34]. From posterior to anterior and through the vibration transmission path, they are ligament 1 (L1) and ligament 2 (L2) associated with tripus, ligament 3 (L3) and ligament 4 (L4) in between ossicles. L1 and L2 connect the tripus from the dorsal edge of its posterior process to the os suspensorium and the base of the rib 4 (outer arm of os suspensorium) respectively. The L3 connects the anterior tip of tripus and posterior end of the intercalarium, whereas L4 connects the intercalarium anterior to the scaphium.

Ligaments L1 to L4 were modelled as linear spring elements following equation (2.1), where ‘*E*’ is the Young's modulus, ‘*S*’ represents the area of cross-section, and ‘*l*’ represents the length of the ligament. The stiffness of the ligament was defined as 1.5 N mm^−1^ calculated from the linear values and dimensions (*E*, *S*, *l*) reported from De Greef *et al.* [43,45] for the lateral mallear and the anterior ligament in humans. The stiffness *K*_lig_ in equation (2.1) of the spring was the force and displacement of the ligaments by De Greef *et al.* [43], which should be the same value in the spring element defined here.

2.1 $$K_{\mathrm{lig}}=\frac{ES}{\ell }.$$

The fourth ossicle and the last of the transmission chain (the anteriormost), the claustrum, is present in zebrafish. It attaches directly to the scaphium. In adult zebrafish, the scaphium is connected to the claustrum by means of a syndesmosis [48], a slightly movable fibrous joint in which bones are joined together by connective tissue. The scaphium and claustrum are therefore the connection of these two ossicles, defined as ‘no separation contact’ in our FE model, which were also considered to move together as a rigid body in a previous study by [34] (Finneran & Hastings [34]. This kind of contact does not allow perpendicular separation between the bones but allows slight movement on the plane on the contact [49].

### 2.4. Boundary conditions in the original model

The connection processes of the intercalarium and scaphium to the vertebrae were described in Alexander [20] and adopted for our models. Since the connection processes are cartilaginous and they are fused to the deeper parts of the socket wall instead of superficial insertion, stiffness of the movements from intercalarium and scaphium to vertebrae is expected to be low. These anatomical features were converted to the FE model for the creation of joints. Joints provide a convenient way to allow for specific types of motion between two entities in ANSYS mechanical analysis. The connections between the intercalarium and scaphium were modelled as spherical joints, which impose constraints on displacements along the three directions of the coordinate axis while permitting rotation along all three axes.

The attachment of the tripus to the centrum is entirely different from that of intercalarium and scaphium. The tripus has an elongate foot attached to the centrum and is expected to behave as a leaf spring. This type of articulation will allow rotation of the tripus in the plane perpendicular to the line of fusion. Moreover, the articulation process of the tripus has a thin sheet of bone, which ends adjacent to the centrum and is layered with cartilage and articulated to the centrum [20]. Therefore, we define the connection of tripus to centrum as a leaf spring (also called semi-elliptical spring), where the displacements are fixed in all the directions and the rotation is free only to the axis of plane of the connection. The rotation to the axis perpendicular to the connection was fixed.

The dorsal surface of the claustrum is connected to the supraneural 2 by means of synchondrosis (cartilaginous joint) [48], and was described as being fused to the first vertebra and thus immobile in zebrafish, functioning to support the wall of the sinus impar atrium [50]. In the FE model, we applied a constraint fixing all the displacements in the dorsal surface of the ossicle.

### 2.5. Lipid cushioning

Through dissections and CT images, we observed that the ossicles of the Weberian apparatus are surrounded by a capsule-like space filled with lipid tissue that provides viscosity and elasticity to the surfaces of the ossicles. This space was also noticed by Alexander [20] and named the saccus paravertebralis. Bird *et al.* [30] recently surveyed the variation of this structure across species of Cypriniformes and confirmed our observation of all Weberian ossicles being surrounded by the saccus paravertebralis in zebrafish. The lipid (loose adipose tissue)-filled space is expected to allow the movement of the Weberian ossicles during vibration with minor impedance. To model the elastic embedment and the viscosity that the loose fat-filled capsule produces, a global dashpot-spring effect is defined in the model for simulating this cushioning effect. The viscous term of the cushioning is defined in terms of Rayleigh damping instead of using a complex modulus. Rayleigh damping is a classical and probably the most common method to build the damping matrix C of a numerical model, under the form C = *α*M + *β*K, where M and K represent the mass and the stiffness of the matrix, and *α* and *β* are the Rayleigh damping coefficients respectively. Rayleigh damping *β* = 0.0001 s and *α* = 0 Hz are assigned because they are the commonly used values in FE models of the human middle ear system [42,45,51,52].

The elastic term of the cushioning is defined using an elastic stiffness surrounding all the outer faces of the ossicles. There is no information of this value in the literature, and we adjusted this value to *K*_cush_ = 0.0025 N mm^−3^. This value fits the results of our harmonic response of FE model with the published mathematical model of Weberian apparatus of *Carassius auratus* [34]. See further details of the adjustment of *K*_cush_ in the electronic supplementary material.

### 2.6. Modal and harmonic analysis

Modal analysis is a process of determining modes, an inherent property of a structure. Modes are characterized in natural frequencies and mode shape during free vibration and defined by the material property and boundary conditions of the structures. The frequency of modes from modal analysis indicates the internal frequencies at which the structure can naturally vibrate. They are also the frequencies at which resonances potentially occur and allow a transfer of energy from one form to another with minimal loss. In human hearing research, the resonance frequency contributes to the hearing sensitivity within hearing frequency ranges [53]. This free vibration analysis is also the basis for the forced vibration harmonic response analysis in next step.

Harmonic analysis predicts the structure's dynamic response including the amplitude, frequency and phases of the oscillatory components, which are subjected to sinusoidally varying loads. For the harmonic analysis, a load must be applied. This analysis has been adopted in previous FE modelling of human middle ear [54]. The Weberian ossicles mechanically couple vibrations of the gas bladder induced by sound to the inner ear fluid, allowing sound to be transduced into electrical signals by the sensory cells there. Therefore, since the aim of the model is analysing how vibrations exerted from the gas bladder transfer to the inner ear, we applied a uniform single-frequency harmonic pressure in the tripus where the gas bladder contacts. As the function of the Weberian ossicles is analogous to that of the middle ear ossicles of terrestrial vertebrates, the pressure applied in the tripus for the harmonic analysis is comparable to the uniform single-frequency harmonic pressure applied as a loading stimulus at the lateral surface of the tympanic membrane in FE models of human hearing [45]. In this case, we solve the modal analysis in the first 50 modes, and then a pressure of 1 Pa is applied to obtain the nominal unitary response in the tip of the tripus where the gas bladder contacts the ossicular chain, and a sweeping of values is done between 10 and 10 000 Hz. This sweeping range well contains the known hearing bandwidth of zebrafish [8–10,12,13,38]. We obtained the results of maximum displacement (μm) and velocity (μm s^−1^) in 200 points evenly distributed in that range. The results of maximum displacement (μm) of the vibrating body would be sufficient to indicate hearing sensitivity at certain frequencies. To compare with published mathematical model [33], we also computed the velocity of the vibrating ossicles, and plotted the amplitude in (μm s^−1^) Pa^−1^.

### 2.7. Models of an ontogenetic series

Ten FE models representing a theoretical ontogenetic series were generated from the original model with geometry constrained and sizes varied. The ossicular chain length ranges from 1 to 10 mm (figure 1), which covers the size of Weberian ossicular chain in majority of otophysan fishes. Material properties and boundary conditions remained the same as in original model. Only some of the parameters of the FE model were adjusted accordingly. Bone properties (Young's modulus *E*, density and Poisson's coefficient) as well as the parameters that define the lipid cushioning (Rayleigh damping parameters *α* and *β* and the elastic term of the cushioning *K*_cush_) are not adjusted because they are values that define the same material. However, the ligament stiffness *K*_lig_ defined in the ligaments needs to be adjusted, since the value of the *K*_lig_ involves size (equation (2.1)) and would change when size and length of the ligaments increase homothetically. The change in the volume of each model and the values adopted for *K*_lig_ can be found in table 1. Other than reconsideration of model parameters, the models were built following the same procedures described above, and the modes of vibration and the harmonic response of the Weberian apparatus under the vibration were obtained.

**Table 1.** Volumes of ossicles and adjustment of *K*_lig_ for the ontogenetic series models.

ossicular chain length (mm)	volume of the tripus (mm^3^)	volume of the intercalarium (mm^3^)	volume of the scaphium (mm^3^)	*K*_lig_ (N mm^−1^)
1	0.002397	0.000172	0.000496	0.58
2	0.019175	0.001374	0.003969	1.16
3	0.064715	0.004638	0.013395	1.73
4	0.153398	0.010994	0.031750	2.31
5	0.299606	0.021473	0.062013	2.89
6	0.517720	0.037106	0.107158	3.47
7	0.822120	0.058923	0.170162	4.04
8	1.227188	0.087955	0.254003	4.62
9	1.747304	0.125233	0.361657	5.20
10	2.396851	0.171787	0.496101	5.78

According to the Buckingham π theorem that is used for dimensional analysis (Kline [55]), different models solved here do not share the same description despite being equivalent or similar. The results are not expected to be a mere projection of changes in size. This is because π-values are not constant along the ontogenetic series. See the electronic supplementary material for details.

To compare the theoretical ontogenetic series and understand the functionality of the Weberian apparatus in transmitting the vibration, we computed two new parameters, the ratio of peak displacement (RPD) and the difference of resonance frequency (DRF). The RPD is the ratio of the peak displacement of the tripus to that of scaphium, which reflects the effectiveness of vibration transmitting (equation (2.2)). The DRF computes the difference between the peak frequencies of the tripus and the scaphium to evaluate whether the vibration keeps the same functionalities (equation (2.3)).

2.2 $$\mathrm{RPD}=\frac{d_{\max}^{\mathrm{tripus}}}{d_{\max}^{\mathrm{scaphium}}}$$

and

2.3 $$\mathrm{DRF}=f_{d_{\max}^{\mathrm{tripus}}}-f_{d_{\max}^{\mathrm{scaphium}}}.$$

Parameter *d* is the amplitude of the displacement in mm and *f* is the frequency in Hz. Values are from table 2.

**Table 2.** From each theoretical ontogenetic FE model: first three modes of free vibration from the modal analysis in the whole Weberian apparatus, as well as Peak displacement and frequency during the harmonic response for the tripus, the intercalarium and the scaphium. Ratio of peak displacement of tripus to scaphium; DRF, difference of resonance frequency of tripus to scaphium. The negligible variation of the RPD indicate the energy transform from tripus to scaphium is equally efficient despite high variations of size. The DRF constantly being at 0 shows resonance frequency of the ossicular chain remains the identical at both ends of the chain which is not affected by size.

ossicular length (mm)	modal analysis			harmonic analysis							
				tripus		intercalarium		scaphium			
	first mode (Hz)	second mode (Hz)	third mode (Hz)	peak frequency (Hz)	peak displacement (μm)	peak frequency (Hz)	peak displacement (μm)	peak frequency (Hz)	peak displacement (μm)	RPD	DRF (Hz)
1	1392	1652	1921	1047.4	4.551	1122.7	0.728	1047.4	0.167606	27.15	0
2	982	1150	1333	911.59	6.191	911.59	0.596	911.59	0.241538	25.63	0
3	802	934	1082	793.41	7.517	766.34	0.556	793.41	0.296167	25.38	0
4	694	807	935	690.55	8.671	690.55	0.552	690.55	0.350993	24.70	0
5	621	720	835	644.24	9.663	644.24	0.522	644.24	0.391182	24.70	0
6	567	657	761	580.52	10.570	560.72	0.513	580.52	0.430232	24.57	0
7	524	608	704	541.59	11.404	541.59	0.511	541.59	0.463882	24.58	0
8	491	568	658	505.26	12.153	505.26	0.504	505.26	0.498711	24.37	0
9	463	535	621	488.03	12.939	488.03	0.512	488.03	0.529568	24.43	0
10	439	508	588	455.29	13.602	471.38	0.499	455.29	0.548178	24.81	0

## 3. Results

### 3.1. Results of the original model

**Figure 2.**
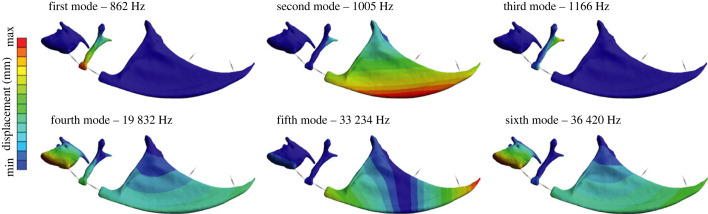
First six modes of free vibration in the Weberian apparatus.

Modal analysis identified the first three modes of vibration at 862, 1005 and 1166 Hz, which are the potential frequencies at which the Weberian ossicular chain could resonate. The next modes of vibration appear in frequencies higher than 19 000 Hz, which are beyond the known hearing range of zebrafish [8–10,12,13,38] and thus not discussed in this study. The first and third modes of the ossicular chain show that peak displacement occurred in the intercalarium, whereas the ventral margin of tripus displays peak displacement at the second mode (figure 2).

**Figure 3.**
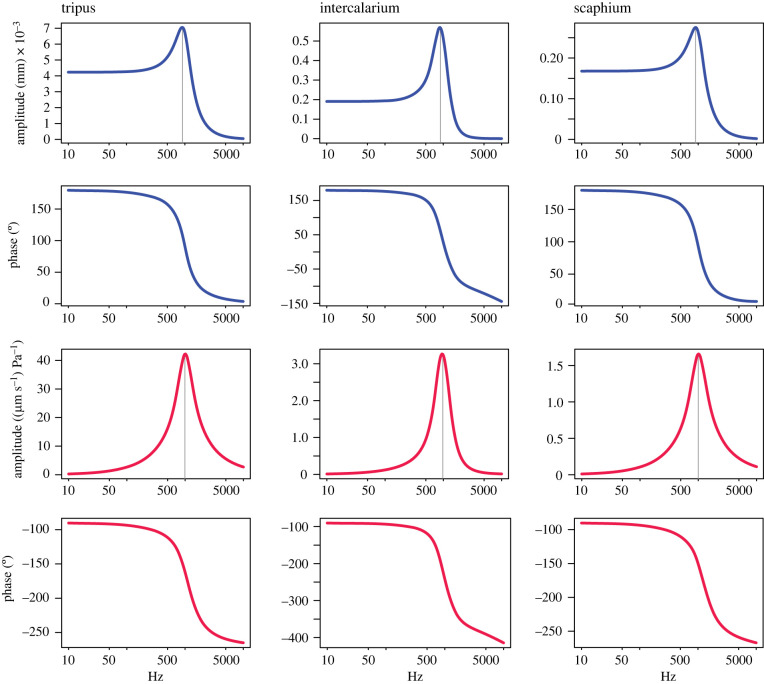
Peak displacement (in blue) and velocity (in red) responses under a cyclic unitary pressure from the gas bladder in the tripus, intercalarium and scaphium.

Results from harmonic analysis shows the response of the Weberian ossicles to the sound pressure exerted by the gas bladder (figure 3). In the displacement response, the resonance appears around 900 Hz (896 Hz) in the tripus, the intercalarium and the scaphium. The peak displacements of tripus, intercalarium and scaphium are about 7, 0.5 and 0.3 µm, respectively. The phases below the resonance frequencies are at 180°, whereas above resonance is at 0° in all three ossicles. In the velocity response, the resonance is visible at around 1000 (1012 Hz in the tripus and scaphium, 944 Hz in intercalarium). The phase's angles are about −90° below the resonance frequencies and close to −270° (or +90°) above resonance in the three ossicles.

### 3.2. Theoretical ontogenetic series

**Figure 4.**
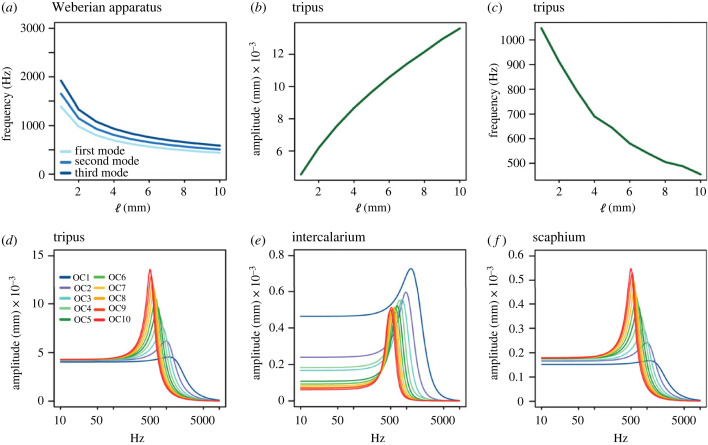
Changes observed in the FE models when changing the length (ℓ) of the ossicular chain (OC) in (*a*) first three modes of vibration, (*b*) peak displacement in the tripus, (*c*) frequency of resonance in the tripus, and harmonic response in the (*d*) tripus, (*e*) intercalarium and (f) scaphium. Different results from corresponding ossicular chain length in harmonic response are indicated by the legend OC.

Results of the modal and the harmonic analysis in the 10 FE models showed inverse relationship between frequencies and sizes. Given the same geometry, the modes of free vibration (figure 4*a*) show negatively related with the sizes. While the amplitudes of the vibration are positively correlated with sizes (figure 4*b*), the frequency of resonance in the harmonic analysis exhibits the opposite trend (figure 4*c*). The correlations are nearly linear. The first three modes with corresponding ossicular displacements obtained from the modal analysis are shown in electronic supplementary material, figure S3.

The harmonic response shows that maximum amplitudes and resonance frequencies gradually shift along size (figure 4*d–f*). Patterns of the curves are nearly identical in tripus and scaphium with same values of resonance frequencies and different level of amplitude (figure 4*d,f*). Despite these changes, the values for the RPD and DRF of the first (tripus) and last ossicle (scaphium) of the transmission chain are practically kept constant along the ontogeny with little variation in the RPD (table 2). The negligible variations of the RPD indicate the energy transform from tripus to scaphium is equally efficient despite varied sizes. The DRF constantly being at 0 shows resonance frequency of the ossicular chain remains identical at both ends of the chain, which is not affected by size. The resonance frequencies of intercalarium (figure 4*e*) gradually change with size, but amplitude of peak displacements does not vary along the frequency. Noticeably, the overall transmission of the vibration was not affected by the extreme behaviour of intercalarium.

### 3.3. Model verification

The velocity response results agree reasonably well with the mathematical predictions and experimental data of [34]. The results are showing that Weberian ossicles are in phase with each other. The phase relationships confirm the expected operation of the Weberian apparatus: positive pressure causes inward radial motion of the anterior gas bladder, forward rotation of the ossicles and fluid flow into the sinus impar [34]. The quality of the agreement verified the FE model for further use.

There have been several comprehensive studies that produced audiograms of zebrafish using various methods, including electrophysiological and psychological-behavioural approaches. While there are variations in the hearing thresholds and frequency ranges tested in these studies, they generally agree on one important aspect—the most sensitive hearing frequency of adult zebrafish, which is consistently found to be around 800 Hz (table 3). In our study, we predict the peak frequency based on the resonance frequency of the Weberian ossicle chain to be 900 Hz. Notably, this particular frequency has not been directly tested in any of the published audiograms of zebrafish. As a result, we currently lack direct evidence to confirm whether there is a discrepancy between our prediction and the actual best hearing frequency observed experimentally. However, it is essential to highlight that our predicted frequency of 900 Hz falls well within the reported best hearing range of zebrafish and is close to the widely tested and documented frequency at 800 Hz. Considering this proximity and the fact that it falls within the best hearing range, we cautiously consider our model to be verified.

**Table 3.** Published hearing sensitivity frequency of wild-type zebrafish. ABR, auditory brainstem response (historical term of AEP). AEBR, acoustically evoked behavioural response. AEP, auditory evoked potentials.

testing methods	sample size	testing range (Hz)	best hearing range (Hz)	best hearing frequency (Hz)	reference
ABR	31	100–4000	800–1000	800	[9]
ABR	10	100–4000	600–1000	800	[8]
AEBR	n/a	100–1200	800	800	[38]
AEP	35	100–8000	600–1000	600	[10]
AEP	39	115–4500	800–1850	800	[12]

## 4. Discussion

A quantitative understanding of the mechanical behaviour of the conductive pathway (external and middle ear, as well as their functional analogues) is crucial for advancing our knowledge of the co-evolution of organisms and their hearing, as well as disorders of hearing in humans. Mathematical and biomechanical modelling serve as a powerful tools in this quest, enabling us to explore and investigate the function and evolution of auditory system of vertebrates. The complex and irregular geometry of conduct hearing apparatuses (e.g. Weberian apparatus), along with a wide range of sizes and displacements involved, makes understanding the mechanical behaviour of the hearing apparatus a challenging task [56]. However, the emergence of high-resolution μCT imaging has revolutionized our ability to visualize and model the detailed anatomy of these structures three-dimensionally [32]. The development of computing power and biomechanical software using finite element methods and reconstruction of natural objects allows modelling of the physical interactions and relationships among multiple component parts with widely varying sizes and properties [57]. The combination of high-resolution CT imaging and advanced biomechanics simulation tools has opened new avenues for realistically modelling the function of conductive hearing apparatus and investigating their mechanical behaviour, which is also the methodology basis of this study.

Finite element methods have been extensively applied to study sound conduction of tympanic ears presented in tetrapods, with a particular focus on the human ear [45,53,54,58–63]. Comparative studies on non-human vertebrates are still limited on cat [64], gerbil [65], chickens [66], mallard [67], whale [68] and mouse [69,70]. This study represents the first finite-element modelling and the first modelling on conductive hearing pathway of atympanic ear. Comparative studies on non-human vertebrate hearing are invaluable in providing insights into the structural and functional divergence and convergence that have occurred through evolution [71]. This research expands on known anatomy and function, and provides potential methodology and parameters to investigate hearing mechanisms across different species and ear types. It may also serve as instrumental guiding for experiments using zebrafish as a model organism for hearing research.

It has long been observed that ‘hearing generalist’ fish exhibit sensitivity to particle motion, while ‘hearing specialist’ fish can detect sound pressure in addition to the particle motion [72]. The ability to sense sound pressure in fish has been linked to the presence of gas bladders and air bubbles [73]. The size of gas bladder may affect hearing [36], and the resonance frequency may contribute to hearing sensitivity [74]. Furthermore, it has been hypothesized that these gas-filled organs in fish serve as acoustic transformers, converting sound pressure into particle motion. This transformed motion is then detectable by the otolith organ in the inner ear of fish [75]. Finite element methods have been employed to model the responses of otoliths to direct sound waves, revealing complex movements in response to varying frequencies and directions of sound [76,77]. It is important to note that the efficacy of the converted input of sound pressure relies on the conductive pathway that delivers the sound stimuli. Fish with a direct connection between the gas bladder and the inner ear exhibit a notably greater hearing range compared with those with only a gas bladder [78]. Otophysans, the group of fish accounting for nearly two-thirds of freshwater fish species, possess such a direct connection facilitated by the Weberian apparatus. However, the exact functionality of this conductive pathway is not yet fully understood.

Resembling all otophysan fishes, Weberian apparatus of zebrafish (*Danio rerio*) largely increase the hearing range through transmitting the sound pressure-induced vibration of gas bladder to the inner ear [37], in addition to sound sources via particle motion in the near field. As soon as the Weberian apparatus is developed and the connection between gas bladder and inner ear established, hearing range of larval fish is the same as that in adult fish [9]. While the coupled motions of the otolith have been experimentally studied and impressively visualized [79], the transmitting process of their connection (the Weberian apparatus) remains unclear. Merely a mathematical model has attempted to reveal the mechanism of sound transmission through Weberian apparatus [34]. The FE model and harmonic analysis of this study use biomechanical methods to understand the functional process of Weberian apparatus. Our model and analysis predict correct amplitude and phase relationships in the zebrafish Weberian ossicles during sound transmission. It demonstrates that the Weberian apparatus acts as a spring-transmitter at audible frequencies that allows to couple the gas bladder motion to the sinus impar and then the inner ear. Different from the mathematical model prediction, the peak displacement is gradually reduced through the ossicle chain. The damping pattern in our FE model is more aligned to the experimental data of *in vivo* amplitude and phase response using non-contact ultrasonic measurement methods than mathematical model in the same publication [34], and therefore is probably more realistic.

Our model and analysis have revealed a couple of functionally important features of the Weberian apparatus. First, the loose connective tissue-filled and capsule-like paravertebral and retroperitoneal space—saccus paravertebralis—is essential for the function of the Weberian ossicles. It is modelled as a global dashpot-spring in this study, which not only provides a space to allow ossicles vibrate, but also provides lipid cushioning (damping effect). As a result, it significantly impacts the mechanical behaviour of Weberian ossicles when compared with middle ear ossicles of tetrapods that vibrate in an air-filled tympanic cavity. Our findings underscore the importance of the saccus paravertebralis in shaping the harmonic response within a reasonable range of frequencies and displacements. Second, shape and size of the intercalarium may have less effect than that of other ossicles on the mechanic behaviour of the ossicle chain. In the isometric series of ossicle chain ranged from 1 to 10 mm, while both ends of the chain (tripus and scaphium) were consistent on resonance frequency, the resonance frequency of intercalarium may be out of range (table 2). If it is true, the intercalarium may display higher function plasticity and morphological variations than the rest of the ossicles. Last but not least, from results of our simulated isometric ontogeny series of Weberian apparatus with constrained geometry, the change of size shifts the resonance frequency, but it does not affect the biomechanical behaviour of the ossicular chain. In the other words, we predict that the best hearing frequency may be lowered but sound transmission efficiency of the apparatus does not change if the fish is undergoing isometric ontogeny.

Higgs *et al.* [8] recorded the hearing threshold of zebrafish at different sizes using physiology apparatus and auditory evoked potential response at 100, 200, 400, 600, 800, 1000, 2000 and 4000 Hz, respectively. They found that there was a significant effect of frequency on threshold (*p* < 0.001) in all tested fishes. Although there was no statistically significant difference found in auditory threshold between the three size classes of fish, the peak frequencies are noticeable from the audiogram plotted against fish size (Higgs *et al.* [8], Figure 9*a*). Zebrafish at size 25–34 mm have lowest threshold at 1000 Hz and the group of 35–44 mm at 800 Hz, whereas the class in 45–50 mm range shows lowest threshold at both 400 and 800 Hz. In the other words, the young juveniles of zebrafish have peak sensitivity at 1000 Hz, larger individuals have peak sensitivity at 800 Hz, and the fully grown adult fish's peak sensitivity is even lower if not the same. The peak frequencies of the three size classes are negatively correlated with size. This pattern from actual measurement is agreed well by the trends predicted by our simulated isometric series of FE models, which means the results obtained in our isometric ontogenetic series can explain observed audiogram in actual zebrafish ontogenetic sequences. These findings suggest that the functionality of the Weberian apparatus may be weakly influenced by possible allometric changes in Weberian ossicles through zebrafish ontogeny. If any other otophysan fish does not present allometry in Weberian ossicles or functionality weakly affected by allometry resembling zebrafish, a shifting of hearing sensitivity towards lower frequency through ontogeny should be observed.

In conclusion, this study presents a pioneering framework for modelling the function and mechanism of the conductive pathway in non-tetrapod and atympanic ears. While the model provides valuable insights on hearing sensitivity estimation, it is important to acknowledge that it is not without limitations. Further advancements and improvements are necessary to enhance its accuracy and applicability. The next crucial step in this research would involve a more comprehensive validation using laser vibrometer measurements. This would provide precise and quantitative data to validate the model's predictions and improve its reliability. Additionally, there is potential to expand the model's scope by incorporating the gas bladder and sinus impar into the equation. This extension would allow for a more holistic understanding of the system and enable the modelling of otolith motion under the influence of fluid dynamics within the sinus impar and inner ear. This model also has potential to further elucidate whether the Weberian apparatus in different otophysan species of varied Weberian apparatus morphologies and sizes are comparable in terms of biomechanical performance. Finally, the model could also be used to explore changes in function that occur with ageing, providing valuable insights into the dynamic nature of the system.

## Data Availability

The CT images and 3D mesh are deposited at MorphoSource (morphosource.org, Media ID 000562737 and 000563167). https://www.morphosource.org/concern/media/000563167 https://www.morphosource.org/concern/media/000562737 The data are provided in electronic supplementary material [80].
